# Evaluation of MHC class I peptide binding prediction servers: Applications for vaccine research

**DOI:** 10.1186/1471-2172-9-8

**Published:** 2008-03-16

**Authors:** Hong Huang Lin, Surajit Ray, Songsak Tongchusak, Ellis L Reinherz, Vladimir Brusic

**Affiliations:** 1 Cancer Vaccine Center, Dana-Farber Cancer Institute, Harvard Medical School, Boston, MA, USA; 2 Department of Mathematics and Statistics, Boston University, Boston, MA, USA; 3 School of Land, Crop and Food Sciences, University of Queensland, Brisbane, Australia

## Abstract

**Background:**

Protein antigens and their specific epitopes are formulation targets for epitope-based vaccines. A number of prediction servers are available for identification of peptides that bind major histocompatibility complex class I (MHC-I) molecules. The lack of standardized methodology and large number of human MHC-I molecules make the selection of appropriate prediction servers difficult. This study reports a comparative evaluation of thirty prediction servers for seven human MHC-I molecules.

**Results:**

Of 147 individual predictors 39 have shown excellent, 47 good, 33 marginal, and 28 poor ability to classify binders from non-binders. The classifiers for HLA-A*0201, A*0301, A*1101, B*0702, B*0801, and B*1501 have excellent, and for A*2402 moderate classification accuracy. Sixteen prediction servers predict peptide binding affinity to MHC-I molecules with high accuracy; correlation coefficients ranging from r = 0.55 (B*0801) to r = 0.87 (A*0201).

**Conclusion:**

Non-linear predictors outperform matrix-based predictors. Most predictors can be improved by non-linear transformations of their raw prediction scores. The best predictors of peptide binding are also best in prediction of T-cell epitopes. We propose a new standard for MHC-I binding prediction – a common scale for normalization of prediction scores, applicable to both experimental and predicted data. The results of this study provide assistance to researchers in selection of most adequate prediction tools and selection criteria that suit the needs of their projects.

## Background

Vaccines are the most effective immunologic intervention in controlling infectious disease [1] and offer a great promise for control of emerging infectious disease, cancer, allergies, and autoimmunity [2]. Peptide-based vaccines offer means for safe and precisely-directed immune intervention; more than 30 peptide-based vaccines are currently under development, including several that are in phase III clinical trials [3]. Most of these vaccines contain various forms of pathogen-derived or tumor-associated antigens. Various strategies for formulation (*e.g*. cells, whole antigens, subunits, or peptides) as well as for delivery systems (*e.g*. dendritic cells, other antigen presenting cells, nanoparticles, recombinant viruses, proteins, and peptides) have been explored in vaccine development [4]. Immunogenic epitopes, the basic immunogenic units within protein antigens, can be used for precise initiation, regulation and control of immune responses [3]. Epitope-based vaccines formulations include peptides (B cell or T-cell); carbohydrates; epitope-coding DNA or RNA; or combinations thereof. A targeted strategy for vaccination focuses on a small number of key antigens and excludes components that are irrelevant (*e.g*. self-proteins on cancer cells) or have capacity to enhance infection or tumor growth [3].

T-cell epitopes are peptides that induce immune responses when bound by major histocompatibility complex (MHC) molecules and presented on the cell surface for recognition by T-cells of the immune system. Peptides derived from degradation of internal proteins that bind MHC-I molecules are recognized by cytotoxic T lymphocytes (CTL). Peptides derived from degradation of external proteins internalized by the antigen presenting cells and bound by MHC class II molecules are recognized by T-helper cells (Th). The development of multivalent vaccines that enable efficient priming, long-lasting and high magnitude CD8^+ ^T-cell immunity is a major direction in the current vaccine research [5]. CTL epitopes induce specific responses against infected or malignant cells, while Th epitopes initiate and regulate immune responses.

Antigens from pathogens or tumors represent suitable targets for immunotherapies and vaccines. Synthetic peptides offer advantages for therapeutic use [6]: they are easy to produce even for a clinical grade, are free from pathogen contamination, have minimal oncogenic potential, and are chemically stable. Peptide epitopes have been used in various formulations of vaccines [7-9]. While some successes of epitope-based cancer vaccines have been reported [10-12], the clinical applications of epitope-based vaccines lag behind and the correlation between responses to T-cell epitopes and clinical outcomes has not been established [13,14]. The formulations for cancer immunotherapies include tumor-specific targets, immune response enhancers, and immune evasion suppressors [15]. Recent clinical studies indicate that high level of tumor infiltration by activated CD8^+ ^T-cells combined with a low number of regulatory T-cells (Treg) is a significant positive prognostic factor for patient survival in cancers [16-19].

Identification of MHC-binding peptides and their subset of T-cell epitopes helps improve our understanding of specificity of immune responses. It is important for discovery of vaccines and immunotherapies [3,6,20]. Tens of thousands of protein variants have been characterized in viruses, such as HIV, influenza, or dengue. The numbers of bacterial, fungal, and parasite antigens are even larger. Several hundred of tumor-related antigens and their variants have been reported [21,22]. More than two thousand variants of human MHC (HLA) have been characterized to date [23]. Given the significant number of antigens and their variants, and a large number of HLA variants, systematic experimental testing of binding capacity of these peptides is impractical. A number of computational methods have been developed to facilitate the identification of MHC binding peptides [24-26]. More than thirty prediction servers have been developed and are accessible via the Internet. These methods use a variety of statistical and machine learning approaches making computational pre-screening of antigens for CTL epitopes a standard approach in epitope-mapping studies. However, with so many choices of prediction servers, new questions have arisen: how to select the best server for a particular HLA allele; can they be used to predict binding affinity of peptides rather than classify into binders and non-binders; and how to use predictions to identify T-cell epitopes amongst HLA ligands? Lack of standards for the development of MHC-I binding predictors resulted in servers that show differences in predictions values and the wide scale of prediction values. Comparisons of methods for prediction of MHC-binding peptides have been reported, indicating high accuracy of binding predictions [27-29]. Predictions of T-cell epitopes, which are a subset of MHC-binders are less accurate and more difficult to model than peptide binding predictions. In a recent study using HLA class I (HLA-I) transgenic mice, 40 candidate T-cell epitopes were identified from computational screening of some 2,900 peptides. Of these 21 were identified as T-cell epitopes and 17 were high-affinity HLA-binders [30]. A new generation of predictive models that combine predictions of multiple antigen processing and presentation steps: HLA binding, peptide binding to transported associated with antigen processing (TAP) and predictions of proteasomal cleavage have been developed (reviewed in [31]). While combination of HLA predictions and TAP predictions offers improvement of predictions in some cases [32], it eliminates TAP-independent peptides from further analysis, such as those produced by vacuolar [33], lysosomal [34], or endosomal [35], among others, pathways. Proteasomal cleavage predictions are of much lower accuracy [32] than HLA-binding or TAP-binding predictions; proteasomal cleavage methods have not yet been adequately validated [31]. The utility and the mode of usage of combined predictors are yet to be determined. In the meantime, HLA-binding predictions remain the most useful computational tools for mapping of HLA ligands and T-cell epitopes.

Peters *et al*. [28] developed a community resource benchmarking prediction on a dataset comprising of 48,828 quantitative peptide-binding affinity measurements. However, a large fraction of this dataset has already been employed by some groups to develop their prediction servers, which may invalidate the comparison with those that did not employ this dataset. Trost *et al*. [29], on the other hand, compared the performance of sixteen servers, and combined predictions by a number of tools into a more accurate combined method. Because of the lack of adequate independent test sets, the comparison studies performed to date have been based on assessing predictive performance using pre-defined sets of peptides, rather than full-overlapping studies of complete antigens. In this study we compared the performance of 30 servers by first normalizing the predictions to a common scale and then assessing the performance using the data from a full-overlapping binding study of 9-mer peptides to seven HLA-I molecules. These peptides were derived from a tumor antigen and from a fragment of a viral antigen. We compared all the servers to find whether any of them produce identical predictions. The main part of the study explored the classification (prediction into binders and non-binders) *vs*. peptide binding affinity prediction capabilities of these servers. We analyzed their prediction performances on two sets of well-defined T-cell epitopes. Finally we explored the non-linear issues of post-processing the prediction values as possible means for improving predictions.

## Results

### Classification

While not all of these servers were designed for the specific purpose of peptide binding predictions, all of them have peptide binding predictions implemented as specific modules. For example MAPPP and ProPred1 predict multiple steps of antigen processing, MULTIPRED predicts peptide binding to HLA supertypes, and BIMAS predicts peptide binding as half-time dissociation (off-rate). Some servers have advanced options, for example MHCPred enables the specification of anchor positions. For this analysis we used the simplest prediction method available at each server. After performing all predictions using the test set, we first calculated the Pearson correlation coefficient for all the servers and found that MAPPP (BIMAS) and MAPPP (SYFPEITHI) showed identical predictions (r = 1) to BIMAS and SYFPEITHI, respectively. The ProPred1 and BIMAS predictions showed r ≥ 0.998 for six HLA-I molecules, r = 0.25 for B*0702, while B*1501 were not available in ProPred1. This was as expected because BIMAS and SYFPEITHI matrices were adopted by MAPPP servers and BIMAS matrices in ProPred1 as HLA-I binding prediction tools. We therefore excluded MAPPP and ProPred1 from further analysis. The numbers of the servers we studied were: A*0201 – 27; A*0301 – 26; A*1101 – 25; A*2402 – 17; B*0702 – 23; B*0801 – 19; and B*1501 – 12. The mutual analysis of predictors by calculating correlation coefficient indicates that these predictors are independent, and predict different subsets of HLA-I binding peptides. These predicted sets are largely overlapping for predictors that employ similar prediction algorithm and show very high accuracy, for example IEDB_ANN and NETM_ANN where r = 0.912.

**Figure 1:**
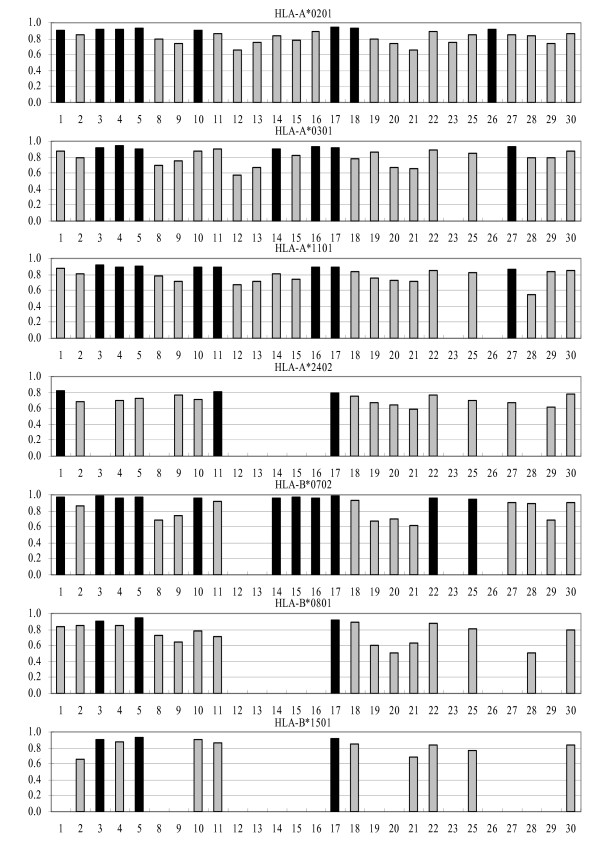
**A_ROC _values of predictions using the combined test set for the 27 servers**. Black bars designate predictors showing the best performance. Vertical axes show the value of A_ROC _while horizontal axes show numbers designating individual servers, as shown in Table 4.

The analysis of classification accuracy (binders vs. non-binders) was performed using the cutoff of 30 (measured binding affinity of ≥ 30% of the binding affinity of a positive control) for binders, while other peptides were considered as experimental non-binders. In total 147 individual predictors were tested of which 39 showed excellent, 47 good, 33 marginal, and 28 poor performance. The A_ROC _values of these predictions are shown in Figure 1.

**Figure 2:**
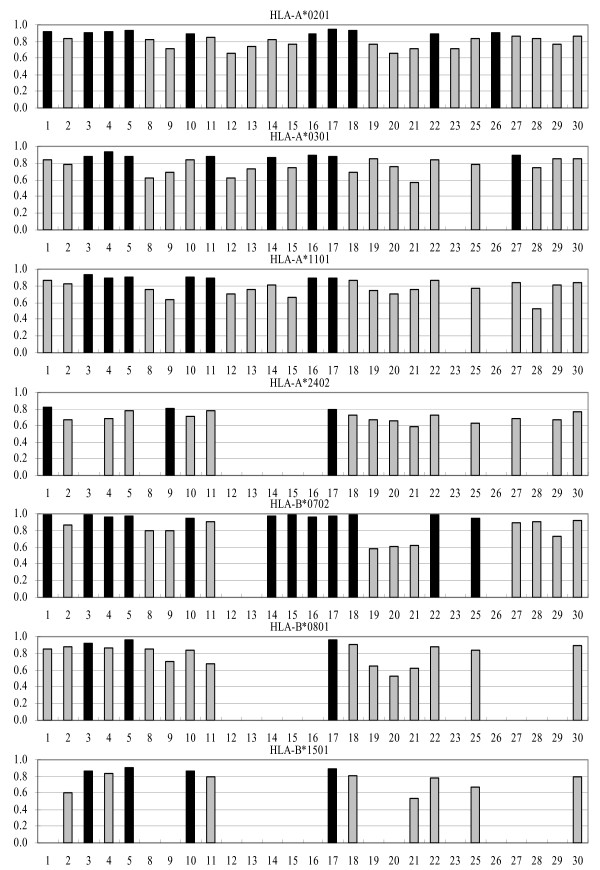
**A_ROC _values of predictions using survivin test set for the 27 servers**. Black bars designate predictors showing the best performance. Vertical axes show the value of AROC while horizontal axes show numbers designating individual servers, as shown in Table 4.

**Figure 3:**
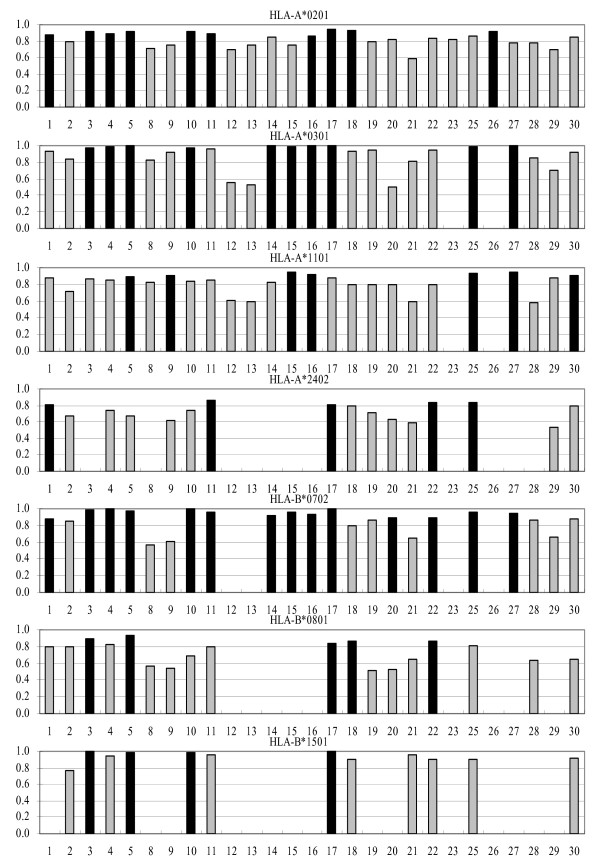
**A_ROC _values of predictions using CMV construct test set for the 27 servers**. Black bars designate predictors showing the best performance. Vertical axes show the value of AROC while horizontal axes show numbers designating individual servers, as shown in Table 4.

We also performed the analysis of survivin test set and CMV construct test set and the results were very similar to the combined set predictions (Figures 2 and 3). The intersection values of sensitivity/specificity plots are consistent with the A_ROC _results. By HLA molecule, the best predictors are for B*0702, where 65% showed excellent classification properties, while approximately 30% of predictors for A*0201, A*0301, and B*1501, and 16% for A*1101 and B*0801 showed excellent classification. The classification accuracy for A*2402 is lower than for other HLA molecules in this study: 18% of predictors showed good classification properties, and the rest showed marginal or poor performance.

The best prediction server across all HLA molecules in this study is NETM_ANN, closely followed by IEDB_ANN and IEDB_SMM. MHCI_MM, MHCI_SM, MULTI_SVM and SVMHC_M also perform well. The best predictors we recommend for classification prediction are shown in Figure 1 as black bars.

### Prediction of binding affinity

**Figure 4:**
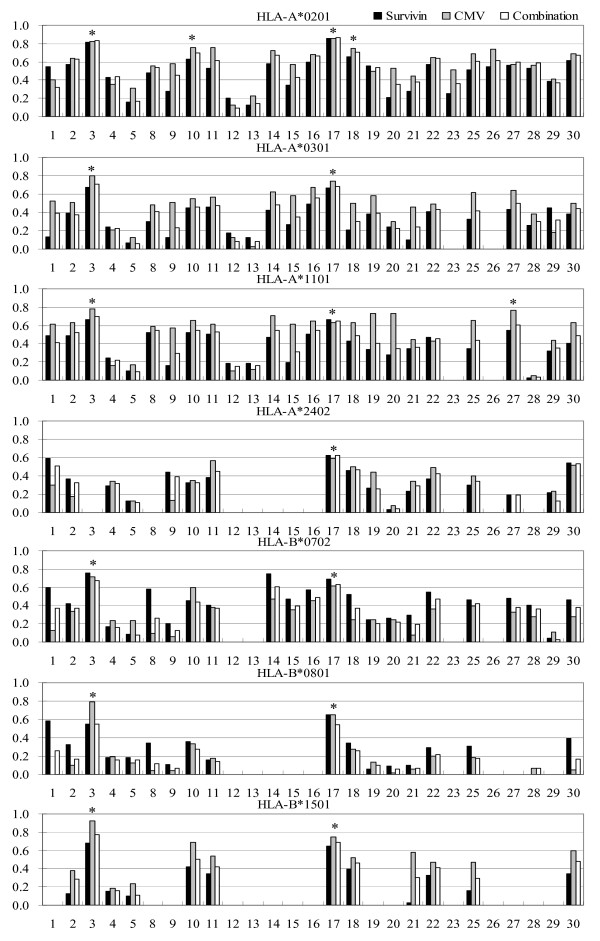
**The correlation coefficients of 27 servers for three datasets**. Black bars for survivin, gray bars for the CMV construct, and white bars for the combined set of peptides. Vertical axis shows the value of correlation coefficients while horizontal axis shows numbers designating individual servers, as shown in Table 4.

Prediction scores from various predictors represent a number of measurable entities. Experimental measurements from the iTopia™ are expressed as the concentration of peptide needed to achieve 50% binding (ED_50 _value) and compared as percentage binding affinity relative to the positive control peptide. For example, the binding scores for BIMAS represent off-rates (minutes), IEDB and NETM_ANN servers represent binding affinity on a nanomolar scales, MHC I server predicts "binding energy", while MULTIPRED server predicts an arbitrary binding score. Large discrepancies are observed even between predictors from the same server. For example the survivin_1–9 _peptide MGAPTLPPA is an experimental binder to A*0201 with estimated 94% affinity relative to the positive control. The respective predictions for IEDB_ANN, IEDB_ARB, and IEDB_SMM are 23441, 365, and 3237 nM, while NETM_ANN predicted value is 8574 nM. Across all predictors a variety of scales and ranges of prediction scores have been observed. Obviously these predictors must be treated as different *in silico *assays and the comparison can be made only by using relative scales of predictions. Using iTopia™ binding assay as the experimental control, we calculated correlation coefficients for all available predictors for three data sets (survivin, CMV construct, and the combined data set). The results show that a high accuracy prediction of peptide binding affinity can be achieved for A*0201 (Figure 4) where IEDB_ANN and NETM_ANN show values of r > 0.8 while A*0201 predictors MHCI_MM, MULTI_ANN, MULTI_SVM, NETM_WM, and SYFPEITHI showed a relatively high correlation coefficient of 0.8 < r < 0.7. The correlation coefficients of predictions for other HLA-I alleles are lower typically 0.6 < r < 0.8 for the best predictors of binding affinity except for B*0801 where the best predictor had r = 0.55. Overall, the best predictors of binding affinity are IEDB_ANN and NETM_ANN. The peptide binding affinities for A*0201 can be predicted *in silico *with high accuracy and, as both the quantity and quality of binding data increases, this will also be achieved for other HLA-I molecules. The best predictors that we recommend for prediction of peptide binding affinity are marked by asterisks (Figure 4).

The peptide binding prediction results across three different datasets show reasonable consistency for indicating that the most predictors generalize well (i.e. predict well across different data sets). For most predictors the prediction accuracy for CMV construct was somewhat higher than for survivin, while the predictions on the combined set were mostly higher than those for survivin and lower than those for CMV construct values. The BIMAS predictions showed low stability in this test, while recommended predictors show high consistency of predictions across the three test sets.

### Non-linear transformations

The predictions of peptide binding classification (Figure 1) show much higher accuracy across different prediction servers than the predictions of binding affinity (Figure 4). For example, the three IEDB predictors and two NetMHC predictors show very similar classification accuracy for A*0201 (Figure 1) while they show significant differences in the prediction of peptide binding affinity where ANN-based predictors are far superior to matrix-based predictors. For each A*0201 predictor, we performed four non-linear transformations and from five sets data selected one that showed the best predictive performance.

**Figure 5:**
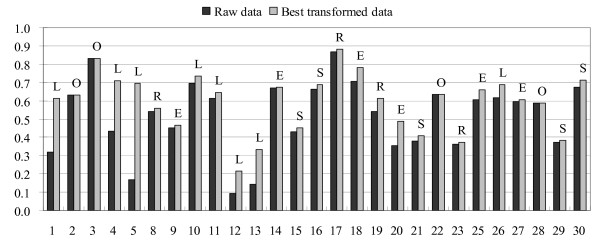
Results of non-linear transformations of the prediction scores for HLA-A*0201. The letters indicate type of transformation that provided the best results: O for original, L for logarithmic, E for exponential, S for square, and R for square root. Vertical axis shows the value of A_ROC _while horizontal axis shows numbers designating individual servers, as shown in Table 4.

The results indicate that the scaling of the output results is a major issue and that it is necessary if linear predictors (matrix-based) are used for prediction of binding affinity. Only four predictors were optimized for output scaling (HLA_LI, IEDB_ANN, PEPC_M, and SVMHC_S), additional fourteen servers showed minor improvements of the correlation coefficient (less than 10% increase relative to the raw predictor output), while the rest of the servers showed sizable improvements (Figure 5). The largest improvements were seen for BIMAS, IEDB_ARB, IEDB_SMM, MHCP_I and MHCP_AA predictors. These results show that most of predictors can be improved by post-processing the prediction outputs through scaling and non-linear transformations. This correction will not affect classification accuracy (binders *vs*. non- binders) since classification is threshold-dependent and the relative order of predictions remains the same as in the raw prediction list. While all four transformations are represented in the improved prediction sets, the largest improvements were achieved by the logarithmic transformation of matrix predictions indicating that in these cases inappropriate formula was used for the definition of matrix coefficients.

### Prediction of T-cell epitopes

We performed prediction of peptide binding with tumor antigen T-cell epitopes and viral epitope sets. Both sets showed similar prediction patterns and we proceeded with the analysis of merged data sets. For each server we predicted the binding affinity of all T- cell epitopes in the merged set and determined the threshold at which approximately 90% of the tested T-cell epitopes were predicted as binders and the threshold at which the first false positive appears at the test set of binders. The higher of the thresholds was used for further analysis for the assessment the number of false positive predictions based on the number performance on the survivin/CMV construct set.

Predictors could be used for different practical purposes. We compared the performance of servers in three scenarios for each predictor (representative results are shown in Tables 1, 2 and 3). These scenarios are represented by the selection of thresholds which corresponding to practical application. The first case is the selection of threshold at which ~90% of T-cell epitopes are predicted as binders; the second threshold predicts correctly the majority of binders (31 of 33); and the third threshold does not allow any non-binders to be predicted as binders. The results clearly show that the superior performance, and thus the selection of the best predictor depend on the practical purpose. For example, NETM_ANN has been judged as the best overall A*0201 predictor (Figure 1 and Figure 4). This server also shows the best performance for thresholds that optimize the selection of T-cell epitopes (Table 1) and the threshold which does not allow false positive (Table 3), but it comes as distant second at the threshold that predicts the vast majority of binders (Table 2). The distinct best predictor for high sensitivity threshold (Table 2) is NHP_CP whose overall performance has been assessed as modest. Overall, considering the balance between false positive and false negative and prediction of T-cell epitopes, NETM_ANN is likely to produce the best result in most cases. The selected thresholds represent the extreme scenarios (high sensitivity, or high specificity predictions). In practical applications, the thresholds will be between these extreme values and costs in terms of false positives and false negatives can be assessed. The higher the sensitivity of prediction, the larger the number of false positives. Conversely, the higher the specificity the lower the number of true positives.

**Table 1:** Prediction performance of selected representative servers in order to correctly predict ~90% of T-cell epitopes

Server	Thr1	TP (binding)	TN (binding)	FP (binding)	FN (binding)	TP (tumor epitopes)	TP (viral epitopes)
BIMAS (A)	2	10	143	0	23	76 (89%)	39 (89%)
MHCP_I (A)	100	31	7	136	2	80 (94%)	40 (91%)
IEDB_SMM (B)	1,000	10	143	0	23	77 (91%)	38 (86%)
NHP_CP (C)	0	31	126	17	2	79 (93%)	40 (91%)
IEDB_ANN (D)	10,000	9	143	0	24	76 (89%)	39 (89%)
MULTI_SVM (D)	5.5	6	141	2	27	79 (93%)	41 (93%)
NETM_ANN (D)	10,000	15	143	0	18	80 (94%)	41 (93%)

**Table 2:** Prediction performance of selected representative servers in order to correctly predict the majority (95%) of binders

Server	Thr2	TP (binding)	TN (binding)	FP (binding)	FN (binding)	TP (tumor epitopes)	TP (viral epitopes)
BIMAS (A)	0.003	31	105	38	2	84 (99%)	44 (100%)
MHCP_I (A)	1,000	31	7	136	2	80 (94%)	40 (91%)
IEDB_SMM (B)	79,000	31	109	34	2	85 (100%)	44 (100%)
NHP_CP (C)	0	31	126	17	2	79 (93%)	40 (91%)
IEDB_ANN (D)	39,000	31	75	68	2	85(100%)	42 (95%)
MULTI_SVM (D)	3.9	31	101	42	2	84 (99%)	44 (100%)
NETM_ANN (D)	40,000	31	113	30	2	85 (100%)	44 (100%)

**Table 3:** Prediction performance of selected representative servers in order to exclude all false positives

Server	Thr3	TP (binding)	TN (binding)	FP (binding)	FN (binding)	TP (tumor epitopes)	TP (viral epitopes)
BIMAS (A)	2	10	143	0	23	76 (89%)	39 (89%)
MHCP_I (A)	10	0	143	0	33	2 (2%)	1 (2%)
IEDB_SMM (B)	1,000	10	143	0	23	77 (91%)	38 (86%)
NHP_CP (C)	0.5	6	143	0	27	76 (89%)	40 (91%)
IEDB_ANN (D)	10,000	9	143	0	24	76 (89%)	39 (89%)
MULTI_SVM (D)	5.8	4	143	0	29	75 (88%)	40 (91%)
NETM_ANN (D)	10,000	15	143	0	18	80 (94%)	41 (93%)

**Figure 6:**
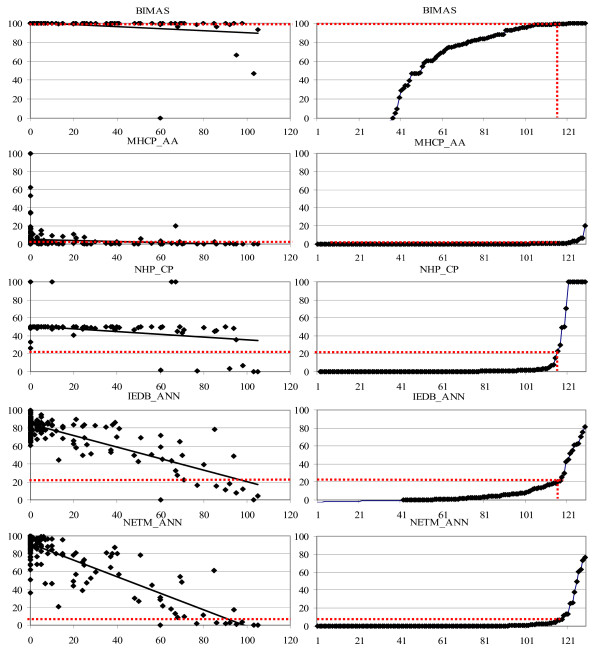
Representative graphs for A*0201 binding predictions on T-cell epitopes and the test peptide. The thresholds marked by broken lines predict approximately 90% of T-cell epitopes and are used for the assessment of false positives and false negatives in binding predictions. Representative examples of predictor groups are shown. The x-axis in the left figure represents experimental scores of test peptides while y-axis represented their scaled predicted scores. The x-axis in the right figure indicates index of sorted list T-cell epitopes while the y-axis represented their scaled predicted binding scores.

Further analysis of results (Figure 6) revealed four main groups of predictors. Group A (BIMAS, MHC_BP, and NHP_ANN) have the majority of predictions clustered at the top of the graph with the nearly horizontal trend line. Although these predictors may provide good prediction of accuracy with carefully selected threshold, however this threshold is difficult to determine. The predictions are of low sensitivity, but relatively high specificity because of a small numbers of TP and FP, and large numbers of TN and FN. Group B (IEDB_SMM, IEDB_ARB, MHCP_I and MHCP_AA) have majority of predictions clustered along the bottom of the graph with the nearly horizontal trend line. Again, these predictions may show good classification accuracy but it is difficult to identify the appropriate threshold. The predictions are typically of high sensitivity and low specificity because of the large number of TP and FP, and small numbers of TN and FN. Group C numbers of (MHC_BPS, MULTI_HMM, NHP_CP, PEPDIST, PREDEP, SVMHC_M, and SVMHC_S) have predictions clustered horizontally or as a cloud with the nearly horizontal trend line. These predictors show moderate accuracy of predictions irrespective of the selected threshold. Finally, the remaining predictors form group D which show the distribution of predictions across the diagonal with a trend line showing slope from non binders to high binders. The accuracy of these predictors is moderate to high with a reasonable balance of TP, TN, FP, and FN. However, these results need to be taken with a note of caution, because some of the T-cell epitopes used for the comparison are likely to be included in the training sets for server development. Nevertheless, it is clear that the servers that are better for prediction of binding affinity are also better in predicting of T-cell epitopes.

In summary, our results have shown that the best predictors of classification also show the best performance in prediction of HLA binding affinity, and prediction of T-cell epitopes, which supports the contention that T-cell epitopes are more likely to be drawn from the highest binding affinity peptides [30,36] and for which quantitative theoretical support has been provided recently [37].

## Conclusions and Discussion

This study shows that major advances have recently been achieved in the field of computational immunology and immunoinformatics. These are mainly the results of the collaborative initiatives that focus on the development of computational infrastructure for immunology, such as IEDB or ImmunoGrid. The availability of large high-quality datasets of HLA ligands and T-cell epitopes and advanced algorithms enabled the development of advanced *in silico *tools that complement experimental research and enable screening collections of pathogen proteomes and large collections of antigens.

We have learnt important lessons about the algorithms that are used to model HLA-peptide interactions. Non-linear algorithms, in particular ANNs appear to offer advantage for prediction of peptide binding affinity. Recently developed algorithms are generally work to be done, since *in silico *assays that match contemporary experimental accuracy are available only for single HLA*0201 9-mer peptides. We have also identified the problems with some prediction methods (Figure 6): group A predictors suffer from low sensitivity and can be improved by re-training their prediction engines with new data, particularly binders; group B suffers from low specificity and these models can be improved by retraining with larger number of non-binders; group C can be further improved by retraining with larger number of training data; while group D can be improved by further improvement of algorithms, while addition of new data is likely to offer only a small gradual improvement for this group. The combination of predictions from high-accuracy predictors is likely to be a major direction for improvement of predictions other than for A*0201 [29]. A large number of predictors, in particular those from groups A and B can be improved by post-processing of raw prediction data, principally non-linear transformation.

Our results also suggest that normalization of outputs by scaling onto a common scale (in this study we used the scale of 0–100) would benefit the field by providing a standard *in silico *scale, which would, in turn, enable mapping of various experimental methods to a common base and fair comparison of the results. In this schema, the negative control peptide maps to 0, while the positive control peptide maps to 100. Binders of higher affinity than the positive control will have binding score greater than 100. The interpretation of the normalized scores is clearer than the raw scores for examples shown in Table 1, 2, 3. Appropriate scaling of outputs also provides practical benefits: a number of predictors that theoretically have good or excellent predictive performance when analyzed in fine detail. However, for those that belong to predictor groups A, B, or C (Figure 6) it is difficult to determine the best threshold for classification predictions because the threshold zone between "good" and "poor" predictions is narrow, rather than wide as in group D predictors. This makes predictors in groups A, B, and C inferior to those in the group D because chances for making poor predictions due to the sub-optimal, or even poor, selection of prediction thresholds by users are high.

The fields of computational immunology and immunoinformatics [25,38] are growing rapidly. Combining experimental and *in silico *methods is essential to address combinatorial problems associated with deciphering immune responses and the applications such as design of vaccines and immunotherapies. While identification of HLA ligands and T-cell epitopes is only a step in the whole process of translation of basic immunology research into clinical applications, it is a prime showcase of significant advances that can be achieved by intelligently combining wet-lab experimentation with mathematical modeling and computation.

## Methods

We identified 30 servers developed by 19 groups that can predict HLA-I binding peptides and are accessible through the Internet (Table 4). The study included several consecutive steps: a) Independent experimentally measured test data sets were identified; b) predictions of peptide binding were made using up to 30 servers (as available for each of the seven HLA-I molecules); c) the predictions of individual servers were compared whether they are identical and "duplicate servers" were removed from further analysis; d) predictions were normalized to the common scale to facilitate comparison of predictive performances; e) classification accuracy (binders *vs*. non-binders) was estimated; f) the accuracy of predicted binding affinities was assessed; g) non-linear transformations of prediction scores were performed for the improvement of predictions. Predictive algorithms used in these studies include: binding matrices [39-50], artificial neural networks – ANN [45,51-54], hidden Markov models – HMM [52], support vector machines [55-58], structure-based model [59,60], partial least square function [61], and peptide-peptide distance function [62].

**Table 4:** List of prediction servers of HLA-I binding peptides, their URLs (as of April 2007), and name abbreviations

**ID**	**Servers**	**Abbreviation**	**URLs**	**Prediction algorithm**	**References**
1	BIMAS	BIMAS	[68]	Matrix	[39]
2	HLA Ligand	HLA_LI	[69]	Matrix	[47]
3	IEDB (ANN)	IEDB_ANN	[70]	ANN	[54]
4	IEDB (ARB)	IEDB_ARB	[71]	Matrix	[48]
5	IEDB (SMM)	IEDB_SMM	[72]	Matrix	[49]
6	MAPPP (Bimas)	MAPPP_B	[73]	Matrix	[43]
7	MAPPP (SYFPEITHI)	MAPPP_S	[74]	Matrix	[43]
8	MHC Binder Prediction	MHC_BP	[75]	Matrix	-
9	MHC-BPS	MHC_BPS	[76]	SVM	[55]
10	MHC-I (Multiple matrix)	MHCI_MM	[77]	Structure-based model	[60]
11	MHC-I (Single matrix)	MHCI_SM	[78]	Structure-based model	[60]
12	MHCPred (Interactions)	MHCP_I	[79]	Partial least square	[61]
13	MHCPred (Amino Acids)	MHCP_AA	[80]	Partial least square	[61]
14	MULTIPRED (ANN)	MULTI_ANN	[81]	ANN	[52]
15	MULTIPRED (HMM)	MULTI_HMM	[82]	HMM	[52]
16	MULTIPRED (SVM)	MULTI_SVM	[83]	SVM	[53]
17	NetMHC (ANN)	NETM_ANN	[84]	ANN	[51]
18	NetMHC (Weight Matrix)	NETM_WM	[85]	Matrix	[44]
19	nHLAPred (ANNPred)	NHP_ANN	[86]	ANN	[53]
20	nHLAPred (ComPred)	NHP_CP	[87]	ANN and Matrix	[53]
21	PepDist	PEPDIST	[88]	distance function	[57]
22	PeptideCheck (Matrix)	PEPC_M	[89]	Matrix	[41]
23	Predep	PREDEP	[90]	Structure-based model	[57]
24	ProPred1	PROPRED	[91]	Matrix	[42]
25	Rankpep	RANKPEP	[92]	Matrix	[41]
26	SMM	SMM	[93]	Matrix	[50]
27	SVMHC (MHCPEP)	SVMHC_M	[94]	SVM	[56]
28	SVMHC (SYFPEITHI)	SVMHC_S	[95]	SVM	[56]
29	SVRMHC	SVRMHC	[96]	SVM	[57]
30	SYFPEITHI	SYFPEITHI	[97]	Matrix	[40]

### Data sets

In this study we used data sets produced by the iTopia™ Epitope Discovery System. The two data sets included the full overlapping study of 134 9-mer peptides spanning the full length of the tumor antigen survivin (Swiss-Prot: O15392) [63] and the 42 peptides spanning a 50 amino acids long construct containing cytomegalovirus (CMV) internal matrix protein pp65 peptides [64].

These studies produced binding data for eight HLA-I molecules (HLA-A*0101, -A*0201, -A*0301, -A*1101, -A*2402, -B*0702, -B*0801, and -B*1501). Only two binders within 176 peptides were identified as -A*0101 binders; this molecule was excluded from further study because of insufficient quantity of test data. For binding/non-binding classification we considered as positives those peptides whose binding affinity was ≥ 30% of the binding affinity of the positive control, as suggested in the iTopia™ technical information. HLA-A*0201 restricted T-cell epitopes have been extracted from the literature and contain 85 well-characterized tumor antigen-related peptides and 44 well-characterized viral T-cell epitopes (see supplemental materials in Additional file 1). Several predictors do not have information on specific genotype alleles but have predictions for serotypes. For instance, the prediction results generated by SMM are actually the binding affinities of peptides to HLA-A2, not exclusively HLA-A*0201. Such approximation may affect their specificity in predicting HLA-A*0201 epitopes to some extent. The data sets used in this study were also deposited in the Dana-Farber Repository for Machine Learning in Immunology [65].

### Predictions and comparisons

The two protein sequences were submitted to the prediction servers and the prediction results were recorded. For each HLA molecule two prediction applications were analyzed: classification into binders and non-binders and prediction of peptide binding affinity. For the assessment of classification accuracy we used the analysis of the area under the ROC curve (A_ROC_) [66].

This curve is a plot of the true positive rate TP/(TP+FN) on the vertical axis *vs*. false positive rate FP/(TN+FP) on the horizontal axis for the full range of the decision thresholds. The values A_ROC_≥0.9 indicate excellent, 0.9>A_ROC_≥0.8 good, 0.8>A_ROC_≥0.7 marginal and 0.7>A_ROC _poor predictions [66]. We also used the sensitivity/specificity plot measure by determining the intersection point of sensitivity and specificity curves for the complete range of thresholds. To assess the accuracy of binding affinity predictions we calculated the Pearson correlation coefficient for experimental measurements X and a prediction series Y for the studied set of peptides:

$$r_{xy}=\frac{\sum \left(x_{i}-\bar{x})(y_{i}-\bar{y}\right)}{\sqrt{\sum {\left(x_{i}-\bar{x}\right)}^{2}\sum {\left(y_{i}-\bar{y}\right)}^{2}}}$$

*x*_*i *_and $\bar{x}$ are experimental individual and average affinities;

*y*_*i *_and $\bar{y}$ are individual and average peptide predictions.

For comparisons of two prediction series the same formula was used except that X and Y represent the results of individual predictions.

To assess the applicability of the prediction servers for identification of T-cell epitopes we performed predictions of peptide binding on two sets (tumor antigen and viral epitopes) of 9-mer HLA-A*0201 restricted T-cell epitopes. We estimated thresholds that identify ~90% of T-cell epitopes as positive predictions (TP) and estimated a number of true positive (TP) false positive (FP), true negative (TN), and false negative (FN) at that threshold using predictions based on the analysis of 176 iTopia™ peptides. Since some of these peptides are well-known, they are likely included in the training sets for individual servers and we should interpret these results only as a guide.

### Scaling and transformations

To enable visual inspection of prediction comparisons, both experimental measurements and predictions were scaled to a common scale from 0 to 100 using linear transformation of the value ranges using the formula for each value for individual peptide:

$$y_{i}^{S}=\frac{y_{i}-y_{\min}}{y_{\max}-y_{\min}}\times 100$$

where $y_{i}^{S}$ is the scaled value, *y*_*min *_is the minimum and *y*_*max *_is the maximum value.

Furthermore we performed non-linear transformations of the raw predicted values from individual servers to assess whether the scaling and normalization issues affect the accuracy of predictions. In statistics, the "power transform", also known as "Box-Cox transform" is used to map data to from one space to another for data stabilization procedures such as reduction of data variation, improvement of the correlation between variables, and improving data distribution [67]. We selected four common non-linear transformations and performed them for each predictor (natural logarithm – L, exponential – E, square – S, and square root – R functions):

$$\begin{array}{l} y_{i}^{Sn}=\ln(y_{i}-y_{\min}+\delta )\\ y_{i}^{Sn}=e^{y_{i}/y_{\max}}\\ y_{i}^{Sn}=y_{i}^{2}\\ y_{i}^{Sn}=\sqrt{y_{i}-y_{\min}} \end{array}$$

where $y_{i}^{Sn}$ is the prediction score for scaled and non-linearly transformed value of raw prediction.

The scaled and transformed predictions were assessed to reveal the predictors have been optimized and those that can be improved by post-processing of prediction values.

## Authors' contributions

HHL carried out the study and drafted the manuscript. SR and ELR participated in the design of the study and critically reviewed the manuscript. ST collected and annotated T-cell epitopes, and prepared the manuscript. VB conceived, designed and coordinated the project, and revised the manuscript. All authors read and approved the final version of the manuscript.

## Supplementary Material

Additional file 1 Data sets used in this study. List of peptide binding affinity and well-characterized T-cell epitopes used in this study. Click here for file
