# Supplementary material for: Evaluation of MHC class I peptide binding prediction servers: Applications for vaccine research
Source: BMC Immunol. 2008 Mar 16;9:8. doi: 10.1186/1471-2172-9-8 (PMC2323361; doi:10.1186/1471-2172-9-8)
Supplement: Additional file 1 — Data sets used in this study. List of peptide binding affinity and well-characterized T-cell epitopes used in this study. [file 1471-2172-9-8-S1.pdf]

Supplemental Table 1. Peptide binding affinities from the BIRC5 (survivin) protein

**Survivin***		Binding scores for various alleles						
Position	Peptide	A*0201	A*0301	A*1101	A*2402	B*0702	B*0801	B*1501
1	MGAPTLPPA	94	1	65	1	2	1	1
2	GAPTLPPAW	4	1	1	74	7	1	0
3	APTLPPAWQ	3	0	0	0	28	2	0
4	PTLPPAWQP	0	0	0	0	0	0	0
5	TLPPAWQPF	69	1	0	110	14	2	90
6	LPPAWQPFL	20	0	0	49	58	2	0
7	PPAWQPFLK	0	28	99	0	2	0	0
8	PAWQPFLKD	0	0	0	0	0	0	0
9	AWQPFLKDH	0	0	0	0	0	0	0
10	WQPFLKDHR	0	0	0	0	0	0	0
11	QPFLKDHRI	8	1	1	11	59	20	0
12	PFLKDHRIS	5	1	0	30	0	11	0
13	FLKDHRIST	77	0	0	0	4	55	40
14	LKDHRISTF	2	0	1	27	1	6	3
15	KDHRISTFK	1	43	66	0	2	1	1
16	DHRISTFKN	0	0	0	0	0	0	0
17	HRISTFKNW	6	7	9	46	3	2	2
18	RISTFKNWP	21	8	13	5	4	2	4
19	ISTFKNWPF	40	3	6	107	16	9	77
20	STFKNWPFL	86	26	54	117	1	3	3
21	TFKNWPFLE	0	0	0	0	0	0	0
22	FKNWPFLEG	0	0	0	0	0	0	0
23	KNWPFLEGC	37	2	1	0	2	1	1
24	NWPFLEGCA	0	0	0	0	0	0	0
25	WPFLEGCAC	0	1	0	33	23	2	1
26	PFLEGCACT	39	0	0	0	0	0	0
27	FLEGCACTP	0	2	0	3	0	1	1
28	LEGCACTPE	0	0	0	0	0	0	0
29	EGCACTPER	0	0	0	0	0	0	0
30	GCACTPERM	0	0	0	0	0	0	0
31	CACTPERMA	0	0	0	0	0	0	0
32	ACTPERMAE	0	0	0	0	0	0	0
33	CTPERMAEA	67	2	1	9	3	3	5
34	TPERMAEAG	0	0	0	0	0	0	0
35	PERMAEAGF	0	0	0	0	0	0	0
36	ERMAEAGFI	0	0	0	0	0	0	0
37	RMAEAGFIH	56	76	100	34	10	3	95
38	MAEAGFIHC	51	4	1	11	3	3	3
39	AEAGFIHCP	0	0	0	0	0	0	0
40	EAGFIHCPT	5	2	1	37	2	4	3
41	AGFIHCPTE	0	0	1	0	0	0	0
42	GFIHCPTEN	0	0	0	0	0	0	0
43	FIHCPTENE	24	3	0	1	1	2	2
44	IHCPTENEP	0	0	0	0	0	0	0
45	HCPTENEPD	0	0	0	0	0	0	0

46	CPTENEPDL	0	0	0	0	0	0	0
47	PTENEPDLA	0	0	0	0	0	0	0
48	TENEPDLAQ	0	0	0	0	0	0	0
49	ENEPDLAQC	0	0	0	0	0	0	0
50	NEPDLAQCF	1	1	1	25	1	1	0
51	EPDLAQCFF	5	0	0	43	3	0	0
52	PDLAQCFFC	0	41	79	40	0	0	0
53	DLAQCFFCF	0	0	0	65	0	0	0
54	LAQCFFCFK	0	0	0	0	1	0	0
55	AQCFFCFKE	3	1	0	0	1	0	19
56	QCFFCFKEL	7	0	0	47	3	0	6
57	CFFCFKELE	0	0	0	70	2	1	0
58	FFCFKELEG	5	1	0	47	1	2	2
59	FCFKELEGW	37	0	0	43	1	1	45
60	CFKELEGWE	0	0	0	0	0	3	80
61	FKELEGWEP	0	0	0	0	0	0	0
62	KELEGWEPD	0	0	0	0	0	0	0
63	ELEGWEPDD	0	0	0	0	0	0	0
64	LEGWEPDDD	0	0	0	0	0	0	0
65	EGWEPDDDP	0	0	0	0	0	0	0
66	GWEPDDDPI	0	0	0	0	0	0	0
67	WEPDDDPIE	0	0	0	0	0	0	0
68	EPDDDPIEE	0	0	0	0	0	0	0
69	PDDDPIEEH	0	0	0	0	0	0	0
70	DDDPIEEHK	0	0	0	0	0	0	0
71	DDPIEEHKK	0	0	0	0	0	0	0
72	DPIEEHKKH	0	0	0	0	0	0	0
73	PIEEHKKHS	0	0	0	0	0	0	0
74	IEEHKKHSS	0	0	0	0	0	0	0
75	EEHKKHSSG	0	0	0	0	0	0	0
76	EHKKHSSGC	0	0	0	0	0	0	0
77	HKKHSSGCA	0	0	0	0	0	0	0
78	KKHSSGCAF	3	1	2	22	4	0	0
79	KHSSGCAFL	26	2	4	86	9	2	2
80	HSSGCAFLS	21	3	18	7	5	3	3
81	SSGCAFLSV	98	4	8	24	6	3	9
82	SGCAFLSVK	7	47	114	18	4	3	2
83	GCAFLSVKK	3	62	100	10	4	3	2
84	CAFLSVKKQ	0	0	0	0	0	0	0
85	AFLSVKKQF	8	1	10	121	5	51	6
86	FLSVKKQFE	20	2	0	19	4	40	2
87	LSVKKQFEE	0	0	0	0	0	0	0
88	SVKKQFEEL	24	3	1	29	42	35	20
89	VKKQFEELT	0	0	0	0	0	0	0
90	KKQFEELTL	0	0	0	0	0	0	0
91	KQFEELTLG	61	2	0	5	4	4	24
92	QFEELTLGE	0	0	0	0	0	0	0
93	FEELTLGEF	5	0	0	39	0	1	6
94	EELTLGEFL	0	0	0	0	0	0	0

95	ELTLGEFLK	5	4	49	11	0	1	0
96	LTLGEFLKL	92	8	44	97	8	3	3
97	TLGEFLKLD	41	1	0	66	0	4	1
98	LGEFLKLDR	0	0	0	0	0	0	0
99	GEFLKLDRE	0	0	0	0	0	0	0
100	EFLKLDRER	0	0	0	0	0	0	0
101	FLKLDRERA	65	8	3	0	1	37	8
102	LKLDRERAK	0	0	0	0	0	0	0
103	KLDRERAKN	38	21	0	11	1	3	0
104	LDRERAKNK	0	0	0	0	0	0	0
105	DRERAKNKI	0	0	0	0	0	0	0
106	RERAKNKIA	0	0	0	0	0	0	0
107	ERAKNKIAK	0	0	0	0	0	0	0
108	RAKNKIAKE	0	0	0	0	0	0	0
109	AKNKIAKET	0	0	0	0	0	0	0
110	KNKIAKETN	0	0	0	0	0	0	0
111	NKIAKETNN	0	0	0	0	0	0	0
112	KIAKETNNK	8	54	86	1	1	3	3
113	IAKETNNKK	4	52	90	1	3	2	3
114	AKETNNKKK	0	0	0	0	0	0	0
115	KETNNKKKE	0	0	0	0	0	0	0
116	ETNNKKKEF	0	0	0	0	0	0	0
117	TNNKKKEFE	0	0	0	0	0	0	0
118	NNKKKEFEE	0	0	0	0	0	0	0
119	NKKKEFEET	0	0	0	0	0	0	0
120	KKKEFEETA	0	0	0	0	0	0	0
121	KKEFEETAK	0	0	0	0	0	0	0
122	KEFEETAKK	0	0	0	0	0	0	0
123	EFEETAKKV	0	0	0	0	0	0	0
124	FEETAKKVR	0	0	0	0	0	0	0
125	EETAKKVRR	0	0	0	0	0	0	0
126	ETAKKVRRA	0	0	0	0	0	0	0
127	TAKKVRRAI	3	2	0	6	42	64	3
128	AKKVRRAIE	0	0	0	0	0	0	0
129	KKVRRAIEQ	0	0	0	0	0	0	0
130	KVRRAIEQL	48	19	1	36	74	26	48
131	VRRAIEQLA	0	0	0	0	0	0	0
132	RRAIEQLAA	0	0	0	0	0	0	0
133	RAIEQLAAM	71	3	25	46	67	5	73
134	AIEQLAAMD	0	0	0	0	0	0	0

* Bachinsky MM, Guillen DE, Patel SR, Singleton J, Chen C, Soltis DA, Tussey LG: **Mapping and binding analysis of peptides derived from the tumor-associated antigen survivin for eight HLA alleles**. *Cancer Immun* 2005, **5**:6.

Supplemental Table 2. Peptide binding affinities from the CMV construct protein

**CMV construct***		Binding scores for various alleles						
Position	Peptide	A*0201	A*0301	A*1101	A*2402	B*0702	B*0801	B*1501
1	MGVIGDQYV	60	0	0	5	5	5	5
2	GVIGDQYVK	35	90	130	10	5	5	5
3	VIGDQYVKV	95	0	35	50	5	5	5
4	IGDQYVKVD	15	0	0	20	5	5	5
5	GDQYVKVDP	10	0	0	15	5	5	5
6	DQYVKVDPV	13	0	0	0	5	35	5
7	QYVKVDPVA	1	0	0	5	5	5	5
8	YVKVDPVAA	60	0	0	0	5	40	50
9	VKVDPVAAL	10	0	0	35	5	5	5
10	KVDPVAALF	70	0	0	110	5	5	70
11	VDPVAALFF	3	0	0	75	5	5	5
12	DPVAALFFF	0	0	0	0	5	0	0
13	PVAALFFFD	3	0	0	5	5	5	5
14	VAALFFFDI	68	0	0	120	5	5	5
15	AALFFFDID	12	0	0	5	5	5	5
16	ALFFFDIDL	60	10	0	10	5	5	30
17	LFFFDIDLQ	5	0	0	10	5	5	5
18	FFFDIDLQA	90	0	0	35	5	5	5
19	FFDIDLQAG	15	0	0	5	5	5	5
20	FDIDLQAGI	50	0	0	5	5	5	5
21	DIDLQAGIL	10	0	0	5	5	5	5
22	IDLQAGILA	25	0	0	60	5	10	5
23	DLQAGILAR	2	0	0	0	5	5	5
24	LQAGILARN	28	0	0	0	5	5	5
25	QAGILARNL	15	0	0	35	5	5	5
26	AGILARNLV	85	0	0	60	5	80	5
27	GILARNLVP	30	0	0	15	10	5	5
28	ILARNLVPM	105	80	35	90	130	200	120
29	LARNLVPMV	80	0	0	10	110	10	10
30	ARNLVPMVA	3	0	0	0	5	5	5
31	RNLVPMVAT	10	0	5	5	5	5	5
32	NLVPMVATV	103	0	0	40	5	5	5
33	LVPMVATVQ	20	0	0	5	0	5	5
34	VPMVATVQG	2	0	0	5	80	5	5
35	PMVATVQGQ	2	0	0	0	5	5	5
36	MVATVQGQN	2	0	0	0	5	5	5
37	VATVQGQNL	25	0	0	80	5	5	5
38	ATVQGQNLK	2	85	120	0	5	5	5
39	TVQGQNLKY	3	70	125	10	5	5	90
40	VQGQNLKYQ	5	0	0	0	5	5	5
41	QGQNLKYQE	2	0	0	5	5	5	5
42	GQNLKYQEF	2	0	5	110	5	5	125

* Movassagh M, Monseaux S, Arnaud L, Necker A, Montero-Julian FA: **Identification of T cell epitopes by iTopia™ epitope discovery system.** *Cytometry A* 2004, **59A**(1):32.

Supplemental Table 3. Well-characterized T-cell epitopes from a selection of tumor-related proteins.

	Tumor protein	Epitope	Length	Allele	Reference
1	BING-4	CQWGRLWQL	9	A2	62
2	p53	LLGRNSFEV	9	A2	31
3	p53	RMPEAAPPV	9	A2	10
4	TRP-2	SVYDFFVWL	9	A2	9
5	TRP-2	TLDSQVMSL	9	A2	32
6	TRP-2-6b	ATTNILEHV	9	A2	64
7	IL13R α2	WLPFGFILI	9	A2	47
8	MAGE-A1	KVLEYVIKV	9	A2	18
9	MAGE-A1	KMVELVHFL	9	A2	34
10	MAGE-A3	FLWGPRALV	9	A2	11
11	MAGE-A3	KVAELVHFL	9	A2	56
12	MAGE-A9	ALSVMGVYV	9	A2	25
13	MAGE-A10	GLYDGMEHL	9	A2	26
14	MAGE-B2	FLWGPRAYA	9	A2	61
15	MAGE C2	ALKDVEERV	9	A2	2
16	NY-ESO-1	SLLMWITQC	9	A2	63
17	NY-ESO-1	QLSLLMWIT	9	A2	1
18	BCR-ABL	FMVELVEGA	9	A2	3
19	BCR-ABL	KLSEQESLL	9	A2	4
20	BCR-ABL	MLTNSCVKL	9	A2	5
21	bcr-abl p210	SSKALQRPV	9	A2	6
22	ETV6-AML1	RIAECILGM	9	A2	7
23	SSX2	KASEKIFYV	9	A2	7
24	Malic Enzyme	FLDEFMEGV	9	A2	7
25	Adipophilin	SVASTITGV	9	A2	8
26	AFP	GVALQTMKQ	9	A2	12
27	CLCA2	LLGNCLPTV	9	A2	13
28	CLCA2	SLQALKVTV	9	A2	14
29	CPSF	KVHPVIWSL	9	A2	14
30	G250	HLSTAFARV	9	A2	19
31	Gnt-V	VLPDVFIRC	9	A2	20
32	Her2/neu	KIFGSLAFL	9	A2	20
33	Her2/neu	IISAVVGIL	9	A2	21
34	Her2/neu	ALCRWGLLL	9	A2	21
35	Her2/neu	ILHNGAYSL	9	A2	22
36	Her2/neu	RLLQETELV	9	A2	23
37	Her2/neu	VVLGVVFGI	9	A2	23
38	Her2/neu	HLYQGCQVV	9	A2	23
39	Her2/neu	PLQPEQLQV	9	A2	24
40	Her2/neu	TLEEITGYL	9	A2	4
41	Her2/neu	ALIHHNTHL	9	A2	27
42	Her2/neu	PLTSIISAV	9	A2	27
43	hTERT	ILAKFLHWL	9	A2	28
44	hTERT	RLVDDFLLV	9	A2	28
45	ML-IAP	SLGSPVLGL	9	A2	28

46	MUC1	STAPPVHNV	9	A2	29
47	MUC1	LLLLTVLTV	9	A2	29
48	MUC2	LLNQLQVNL	9	A2	30
49	MUC2	MLWGWREHV	9	A2	30
50	PRAME	VLDGLDVLL	9	A2	33
51	PRAME	SLLQHLIGL	9	A2	33
52	RAGE1	LKLSGVVRL	9	A2	35
53	SART3	LLQAEAPRL	9	A2	36
54	SART3	RLAEYQAYI	9	A2	37
55	SOX10	AWISKPPGV	9	A2	38
56	WT1	RMFPNAPYL	9	A2	39
57	CEACAM5	YLSGANLNL	9	A2	40
58	CEACAM5	IMIGVLVGV	9	A2	41
59	ep-CAM	GLKAGVIAV	9	A2	42
60	gp100	KTWGQYWQV	9	A2	42
61	gp100	MLGTHTMEV	9	A2	43
62	gp100	ITDQVPFSV	9	A2	44
63	gp100	YLEPGPVTA	9	A2	45
64	gp100	RLMKQDFSV	9	A2	46
65	gp100	RLPRIFCSC	9	A2	48
66	MC1R	TILLGIFFL	9	A2	49
67	MC1R	FLALIICNA	9	A2	50
68	Melan-A	AAGIGILTV	9	A2	51
69	Melan-A	ILTVILGVL	9	A2	51
70	Polypeptide 15	IMLCLIAAV	9	A2	52
71	Tyrosinase	MLLAVLYCL	9	A2	53
72	Tyrosinase	YMDGTMSQV	9	A2	54
73	CASP-5	FLIIWQNTM	9	A2	54
74	HERV-K-MEL	MLAVISCAV	9	A2	55
75	NY-BR-1	SLSKILDTV	9	A2	55
76	NY-MEL-1	VLHWDPETV	9	A2	55
77	MMP2	GLPPDVQRV	9	A2	55
78	RNF43	ALWPWLLMA	9	A2	55
79	Secernin 1	KMDAEHPEL	9	A2	57
80	STEAP1	MIAVFLPIV	9	A2	58
81	707-AP	RVAALARDA	9	A2	59
82	TGFbRII	RLSSCVPVA	9	A2	60
83	FATE	ILLRDAGLV	9	A*0201	15
84	ephA2	IMNDMPIYM	9	A*0201	16
85	ephA2	VLAGVGFFI	9	A*0201	17

*See supplemental references

Supplemental Table 4. Well-characterized HLA-A*0201 restricted T-cell epitopes from a selection of medically important viruses.

	Virus protein	Epitope	Length	Allele	Reference
1	Adenovirus 3 E3	LIVIGILIL	9	A*0201	65
2	EBV BMLF-1	GLCTLVAML	9	A*0201	66
3	EBV EBNA-3	SVRDRLARL	9	A*0201	67
4	EBV-encoded BARFO ORF protein	LLWAARPRL	9	A*0201	68
5	EBV gp350	VLQWASLAV	9	A*0201	69
6	EBV gp85	LMIIPLINV	9	A*0201	69
7	EBV gp85	TLFIGSHVV	9	A*0201	69
8	EBV gp85	SLVIVTTFV	9	A*0201	69
9	EBV LMP-1	YLLEMLWRL	9	A*0201	70
10	EBV LMP-2	CLGGLLTMV	9	A*0201	71
11	EBV LMP-2	LLWTLVVLL	9	A*0201	72
12	HBV DNA pol	GLSRYVARL	9	A*0201	73
13	HBV surface antigen	WLSLLVPFV	9	A*0201	74
14	HBV surface antigen	FLLTRILTI	9	A*0201	73
15	HCMV IE 1	VLEETSVML	9	A*0201	75
16	HCMV pp65	NLVPMVATV	9	A*0201	76
17	HCMV pp65	MLNIPSINV	9	A*0201	76
18	HCMV pp65	RIFAELEGV	9	A*0201	77
19	HCMV pp65	VLGPISGHV	9	A*0201	78
20	HCV-1 env E	SMVGNWAKV	9	A*0201	79
21	HCV-1 MP	DLMGYIPLV	9	A*0201	80
22	HCV-1 NS3	CINGVCWTV	9	A*0201	81
23	HCV-1 NS3	YLVTRHADV	9	A*0201	82
24	HCV-1 NS5	GLQDCTMLV	9	A*0201	83
25	HIV-1 (BRU) gag p17	SLYNTVATL	9	A*0201	84
26	HIV-1 (BRU) gag p24	TLNAWVKVV	9	A*0201	85
27	HIV-1 (BRU) nef	AFHHVAREL	9	A*0201	86
28	HIV-1 (BRU) RT	ILKEPVHGV	9	A*0201	87
29	HIV-1 (BRU) RT	VIYQYMDDL	9	A*0201	88
30	HIV-1 (MAL) gp120	KLTPLCVTL	9	A*0201	89
31	HPV11 E7	RLVTLKDIV	9	A*0201	90
32	HPV16E7	LLMGTLGIV	9	A*0201	91
33	HPV 6b E7	GLHCYEQLV	9	A*0201	90
34	HPV 6b E7	PLKQHFQIV	9	A*0201	90
35	HSV-1 gp C	GIGIGVLAA	9	A*0201	65
36	HSV-2 gp C	GAGIGVAVL	9	A*0201	65
37	HSV-2 UL47	FLVDAIVRV	9	A*0201	92
38	HSV-2 UL47	GLADTVVAC	9	A*0201	92
39	HTLV-1 tax	LLFGYPVYV	9	A*0201	93
40	Influenza A MP	GILGFVFTL	9	A*0201	94
41	Influenza A (PR8) NS1	AIMDKNIIL	9	A*0201	95
42	Vaccinia virus 49.8 kD protein	ILDDNLYKV	9	A*0201	96
43	Vaccinia virus protein H3L	SLSAYIIRV	9	A*0201	97
44	VP1	SITEVECFL	9	A*0201	98

*See supplemental references

Supplemental references

1. Anderson BW, Kudelka AP, Honda T, Pollack MS, Gershenson DM, Gillogly MA, Murray JL, Ioannides CG. Induction of determinant spreading and of Th1 responses by in vitro stimulation with HER-2 peptides. Cancer Immunol Immunother. 2000 Nov;49(9):459-68.
2. Asai T, Storkus WJ, Mueller-Berghaus J, Knapp W, DeLeo AB, Chikamatsu K, Whiteside TL. In vitro generated cytolytic T lymphocytes reactive against head and neck cancer recognize multiple epitopes presented by HLA-A2, including peptides derived from the p53 and MDM-2 proteins. Cancer Immun. 2002 Apr 16;2:3.
3. Azuma K, Shichijo S, Maeda Y, Nakatsura T, Nonaka Y, Fujii T, Koike K, Itoh K. Mutated p53 gene encodes a nonmutated epitope recognized by HLA-B*4601-restricted and tumor cell-reactive CTLs at tumor site. Cancer Res. 2003 Feb 15;63(4):854-8.
4. Barfoed AM, Petersen TR, Kirkin AF, Thor Straten P, Claesson MH, Zeuthen J. Cytotoxic T-lymphocyte clones, established by stimulation with the HLA-A2 binding p5365-73 wild type peptide loaded on dendritic cells In vitro, specifically recognize and lyse HLA-A2 tumour cells overexpressing the p53 protein. Scand J Immunol. 2000 Feb;51(2):128-33.
5. Brossart P, Heinrich KS, Stuhler G, Behnke L, Reichardt VL, Stevanovic S, Muhm A, Rammensee HG, Kanz L, Brugger W. Identification of HLA-A2-restricted T-cell epitopes derived from the MUC1 tumor antigen for broadly applicable vaccine therapies. Blood. 1999 Jun 15;93(12):4309-17.
6. Butterfield LH, Koh A, Meng W, Vollmer CM, Ribas A, Dissette V, Lee E, Glaspy JA, McBride WH, Economou JS Generation of human T-cell responses to an HLA-A2.1-restricted peptide epitope derived from alpha-fetoprotein. Cancer Res. 1999 Jul 1;59(13):3134-42.
7. Buzyn A, Ostankovitch M, Zerbib A, Kemula M, Connan F, Varet B, Guillet JG, Choppin J. Peptides derived from the whole sequence of BCR-ABL bind to several class I molecules allowing specific induction of human cytotoxic T lymphocytes. Eur J Immunol. 1997 Aug;27(8):2066-72.
8. Castelli C, Storkus WJ, Maeurer MJ, Martin DM, Huang EC, Pramanik BN, Nagabhushan TL, Parmiani G, Lotze MT. Mass spectrometric identification of a naturally processed melanoma peptide recognized by CD8+ cytotoxic T lymphocytes J Exp Med. 1995 Jan 1;181(1):363-8.
9. Chaux P, Luiten R, Demotte N, Vantomme V, Stroobant V, Traversari C, Russo V, Schultz E, Cornelis GR, Boon T, van der Bruggen P. Identification of five MAGE-A1 epitopes recognized by cytolytic T lymphocytes obtained by in vitro stimulation with dendritic cells transduced with MAGE-A1. J Immunol. 1999 Sep 1;163(5):2928-36.
10. Chiriva-Internati M, Wang Z, Pochopien S, Salati E, Lim SH. Identification of a sperm protein 17 CTL epitope restricted by HLA-A1. Int J Cancer. 2003 Dec 10;107(5):863-5.
11. Corman JM, Sercarz EE, Nanda NK. Recognition of prostate-specific antigenic peptide determinants by human CD4 and CD8 T cells. Clin Exp Immunol. 1998 Nov;114(2):166-72.
12. Cox AL, Skipper J, Chen Y, Henderson RA, Darrow TL, Shabanowitz J, Engelhard VH, Hunt DF, Slingluff CL Jr. Identification of a peptide recognized by five melanoma-specific human cytotoxic T cell lines. Science. 1994 Apr 29;264(5159):716-9.
13. Fisk B, Blevins TL, Wharton JT, Ioannides CG. Identification of an immunodominant peptide of HER-2/neu protooncogene recognized by ovarian tumor-specific cytotoxic T lymphocyte lines. J Exp Med. 1995 Jun 1;181(6):2109-17.
14. Flad T, Spengler B, Kalbacher H, Brossart P, Baier D, Kaufmann R, Bold P, Metzger S, Bluggel M, Meyer HE, Kurz B, Muller CA. Direct identification of major histocompatibility complex class I-bound tumor-associated peptide antigens of a renal carcinoma cell line by a novel mass spectrometric method. Cancer Res. 1998 Dec 15;58(24):5803-11
15. Gaudin C, Kremer F, Angevin E, Scott V, Triebel F. A hsp70-2 mutation recognized by CTL on a human renal cell carcinoma. J Immunol. 1999 Feb 1;162(3):1730-8.
16. Gnjatic S, Nagata Y, Jager E, Stockert E, Shankara S, Roberts BL, Mazzara GP, Lee SY, Dunbar PR, Dupont B, Cerundolo V, Ritter G, Chen YT, Knuth A, Old LJ. Strategy for monitoring T cell responses to NY-ESO-1 in patients with any HLA class I allele. Proc Natl Acad Sci U S A. 2000 Sep 26;97(20):10917-22.
17. Gueguen M, Patard JJ, Gaugler B, Brasseur F, Renauld JC, Van Cangh PJ, Boon T, Van den Eynde BJ. An antigen recognized by autologous CTLs on a human bladder carcinoma. J Immunol. 1998 Jun 15;160(12):6188-94.
18. Hanada K, Yewdell JW, Yang JC. Immune recognition of a human renal cancer antigen through post-translational protein splicing. Nature. 2004 Jan 15;427(6971):252-6.

19. Huang LQ, Brasseur F, Serrano A, De Plaen E, van der Bruggen P, Boon T, Van Pel A. Cytolytic T lymphocytes recognize an antigen encoded by MAGE-A10 on a human melanoma. J Immunol. 1999 Jun 1;162(11):6849-54.
20. Ito M, Shichijo S, Miyagi Y, Kobayashi T, Tsuda N, Yamada A, Saito N, Itoh K. Identification of SART3-derived peptides capable of inducing HLA-A2-restricted and tumor-specific CTLs in cancer patients with different HLA-A2 subtypes. Int J Cancer. 2000 Nov 15;88(4):633-9.
21. Jager E, Chen YT, Drijfhout JW, Karbach J, Ringhoffer M, Jager D, Arand M, Wada H, Noguchi Y, Stockert E, Old LJ, Knuth A. Simultaneous humoral and cellular immune response against cancer-testis antigen NY-ESO-1: definition of human histocompatibility leukocyte antigen (HLA)-A2-binding peptide epitopes. J Exp Med. 1998 Jan 19;187(2):265-70.
22. Jager E, Hohn H, Karbach J, Momburg F, Castelli C, Knuth A, Seliger B, Maeurer MJ. Cytotoxic T lymphocytes define multiple peptide isoforms derived from the melanoma-associated antigen MART-1/Melan-A. Int J Cancer. 1999 Jun 11;81(6):979-84.
23. Jaramillo A, Narayanan K, Campbell LG, Benshoff ND, Lybarger L, Hansen TH, Fleming TP, Dietz JR, Mohanakumar T. Recognition of HLA-A2-restricted mammaglobin-A-derived epitopes by CD8+ cytotoxic T lymphocytes from breast cancer patients. Breast Cancer Res Treat. 2004 Nov;88(1):29-41.
24. Karanikas V, Colau D, Baurain JF, Chiari R, Thonnard J, Gutierrez-Roelens I, Goffinet C, Van Schaftingen EV, Weynants P, Boon T, Coulie PG. High frequency of cytolytic T lymphocytes directed against a tumor-specific mutated antigen detectable with HLA tetramers in the blood of a lung carcinoma patient with long survival. Cancer Res. 2001 May 1;61(9):3718-24.
25. Kawakami Y, Robbins PF, Wang X, Tupesis JP, Parkhurst MR, Kang X, Sakaguchi K, Appella E, Rosenberg SA. Identification of new melanoma epitopes on melanosomal proteins recognized by tumor infiltrating T lymphocytes restricted by HLA-A1, -A2, and -A3 alleles. J Immunol. 1998 Dec 15;161(12):6985-92.
26. Kawakami Y, Wang X, Shofuda T, Sumimoto H, Tupesis J, Fitzgerald E, Rosenberg S. Isolation of a new melanoma antigen, MART-2, containing a mutated epitope recognized by autologous tumor-infiltrating T lymphocytes. J Immunol. 2001 Feb 15;166(4):2871-7.
27. Kawakami Y, Robbins PF, Wang X, Tupesis JP, Parkhurst MR, Kang X, Sakaguchi K, Appella E, Rosenberg SA. Identification of new melanoma epitopes on melanosomal proteins recognized by tumor infiltrating T lymphocytes restricted by HLA-A1, -A2, and -A3 alleles. J Immunol. 1998 Dec 15;161(12):6985-92.
28. Kawashima I, Hudson SJ, Tsai V, Southwood S, Takesako K, Appella E, Sette A, Celis E. The multi-epitope approach for immunotherapy for cancer: identification of several CTL epitopes from various tumor-associated antigens expressed on solid epithelial tumors. Hum Immunol. 1998 Jan;59(1):1-14.
29. Kessler JH, Beekman NJ, Bres-Vloemans SA, Verdijk P, van Veelen PA, Kloosterman-Joosten AM, Vissers DC, ten Bosch GJ, Kester MG, Sijts A, Wouter Drijfhout J, Ossendorp F, Offringa R, Melief CJ. Efficient identification of novel HLA-A(*)0201-presented cytotoxic T lymphocyte epitopes in the widely expressed tumor antigen PRAME by proteasome-mediated digestion analysis. J Exp Med. 2001 Jan 1;193(1):73-88.
30. Khong HT, Rosenberg SA. The Waardenburg syndrome type 4 gene, SOX10, is a novel tumor-associated antigen identified in a patient with a dramatic response to immunotherapy.Cancer Res. 2002 Jun 1;62(11):3020-3.
31. Kittlesen DJ, Thompson LW, Gulden PH, Skipper JC, Colella TA, Shabanowitz J, Hunt DF, Engelhard VH, Slingluff CL Jr. Human melanoma patients recognize an HLA-A1-restricted CTL epitope from tyrosinase containing two cysteine residues: implications for tumor vaccine development. J Immunol. 1998 Mar 1;160(5):2099-106.
32. Kobayashi T, Lonchay C, Colau D, Demotte N, Boon T, van der Bruggen P. New MAGE-4 antigenic peptide recognized by cytolytic T lymphocytes on HLA-A1 tumor cells. Tissue Antigens. 2003 Nov;62(5):426-32.
33. Konopitzky R, Konig U, Meyer RG, Sommergruber W, Wolfel T, Schweighoffer T. Identification of HLA-A*0201-restricted T cell epitopes derived from the novel overexpressed tumor antigen calcium-activated chloride channel 2. J Immunol. 2002 Jul 1;169(1):540-7.
34. Linard B, Bezieau S, Benlalam H, Labarriere N, Guilloux Y, Diez E, Jotereau F. A ras-mutated peptide targeted by CTL infiltrating a human melanoma lesion. J Immunol. 2002 May 1;168(9):4802-8.

35. Linnebacher M, Gebert J, Rudy W, Woerner S, Yuan YP, Bork P, von Knebel Doeberitz M. Frameshift peptide-derived T-cell epitopes: a source of novel tumor-specific antigens. Int J Cancer. 2001 Jul 1;93(1):6-11.
36. Ma W, Germeau C, Vigneron N, Maernoudt AS, Morel S, Boon T, Coulie PG, Van den Eynde BJ. Two new tumor-specific antigenic peptides encoded by gene MAGE-C2 and presented to cytolytic T lymphocytes by HLA-A2. Int J Cancer. 2004 May 1;109(5):698-702.
37. Maeda Y, Ito M, Harashima N, Nakatsura T, Hida N, Imai N, Sato Y, Shichijo S, Todo S, Itoh K. Cleavage and polyadenylation specificity factor (CPSF)-derived peptides can induce HLA-A2-restricted and tumor-specific CTLs in the majority of gastrointestinal cancer patients. Int J Cancer. 2002 May 20;99(3):409-17.
38. Minev B, Hipp J, Firat H, Schmidt JD, Langlade-Demoyen P, Zanetti M. Cytotoxic T cell immunity against telomerase reverse transcriptase in humans. Proc Natl Acad Sci U S A. 2000 Apr 25;97(9):4796-801.
39. Morioka N, Kikumoto Y, Hoon DS, Morton DL, Irie RF. Cytotoxic T cell recognition of a human melanoma derived peptide with a carboxyl-terminal alanine-proline sequence. Mol Immunol. 1995 Jun;32(8):573-81.
40. Nagorsen D, Keilholz U, Rivoltini L, Schmittel A, Letsch A, Asemissen AM, Berger G, Buhr HJ, Thiel E, Scheibenbogen C. Natural T-cell response against MHC class I epitopes of epithelial cell adhesion molecule, her-2/neu, and carcinoembryonic antigen in patients with colorectal cancer. Cancer Res. 2000 Sep 1;60(17):4850-4.
41. Noppen C, Levy F, Burri L, Zajac P, Remmel E, Schaefer C, Luscher U, Heberer M, Spagnoli GC. Naturally processed and concealed HLA-A2.1-restricted epitopes from tumor-associated antigen tyrosinase-related protein-2. Int J Cancer. 2000 Jul 15;87(2):241-6.
42. Oehlrich N, Devitt G, Linnebacher M, Schwitalle Y, Grosskinski S, Stevanovic S, Zoller M. Generation of RAGE-1 and MAGE-9 peptide-specific cytotoxic T-lymphocyte lines for transfer in patients with renal cell carcinoma. Int J Cancer. 2005 Nov 1;117(2):256-64.
43. Oka Y, Elisseeva OA, Tsuboi A, Ogawa H, Tamaki H, Li H, Oji Y, Kim EH, Soma T, Asada M, Ueda K, Maruya E, Saji H, Kishimoto T, Udaka K, Sugiyama H. Human cytotoxic T-lymphocyte responses specific for peptides of the wild-type Wilms' tumor gene (WT1) product. Immunogenetics. 2000 Feb;51(2):99-107.
44. Okano F, Storkus WJ, Chambers WH, Pollack IF, Okada H. Identification of a novel HLA-A*0201-restricted, cytotoxic T lymphocyte epitope in a human glioma-associated antigen, interleukin 13 receptor alpha2 chain. Clin Cancer Res. 2002 Sep;8(9):2851-5.
45. Ottaviani S, Zhang Y, Boon T, van der Bruggen P. A MAGE-1 antigenic peptide recognized by human cytolytic T lymphocytes on HLA-A2 tumor cells. Cancer Immunol Immunother. 2005 Dec;54(12):1214-20. Epub 2005 Jul 16.
46. Parkhurst MR, Fitzgerald EB, Southwood S, Sette A, Rosenberg SA, Kawakami Y. Identification of a shared HLA-A*0201-restricted T-cell epitope from the melanoma antigen tyrosinase-related protein 2 (TRP2). Cancer Res. 1998 Nov 1;58(21):4895-901.
47. Paschen A, Jing W, Drexler I, Klemm M, Song M, Muller-Berghaus J, Nguyen XD, Osen W, Stevanovic S, Sutter G, Schadendorf D. Melanoma patients respond to a new HLA-A*01-presented antigenic ligand derived from a multi-epitope region of melanoma antigen TRP-2. Int J Cancer. 2005 Oct 10;116(6):944-8.
48. Petersen TR, Buus S, Brunak S, Nissen MH, Sherman LA, Claesson MH. Identification and design of p53-derived HLA-A2-binding peptides with increased CTL immunogenicity. Scand J Immunol. 2001 Apr;53(4):357-64.
49. Rimoldi D, Rubio-Godoy V, Dutoit V, Lienard D, Salvi S, Guillaume P, Speiser D, Stockert E, Spagnoli G, Servis C, Cerottini JC, Lejeune F, Romero P, Valmori D. Efficient simultaneous presentation of NY-ESO-1/LAGE-1 primary and nonprimary open reading frame-derived CTL epitopes in melanoma. J Immunol. 2000 Dec 15;165(12):7253-61.
50. Rodeberg DA, Nuss RA, Elsawa SF, Celis E. Recognition of six-transmembrane epithelial antigen of the prostate-expressing tumor cells by peptide antigen-induced cytotoxic T lymphocytes. Clin Cancer Res. 2005 Jun 15;11(12):4545-52.
51. Rongcun Y, Salazar-Onfray F, Charo J, Malmberg KJ, Evrin K, Maes H, Kono K, Hising C, Petersson M, Larsson O, Lan L, Appella E, Sette A, Celis E, Kiessling R. Identification of new HER2/neu-derived peptide epitopes that can elicit specific CTL against autologous and allogeneic carcinomas and melanomas. J Immunol. 1999 Jul 15;163(2):1037-44. PMID: 10395702

52. Ropke M, Hald J, Guldberg P, Zeuthen J, Norgaard L, Fugger L, Svejgaard A, Van der Burg S, Nijman HW, Melief CJ, Claesson MH. Spontaneous human squamous cell carcinomas are killed by a human cytotoxic T lymphocyte clone recognizing a wild-type p53-derived peptide. Proc Natl Acad Sci U S A. 1996 Dec 10;93(25):14704-7.
53. Rosenberg SA, Tong-On P, Li Y, Riley JP, El-Gamil M, Parkhurst MR, Robbins PF. Identification of BING-4 cancer antigen translated from an alternative open reading frame of a gene in the extended MHC class II region using lymphocytes from a patient with a durable complete regression following immunotherapy. J Immunol. 2002 Mar 1;168(5):2402-7.
54. Salazar-Onfray F, Nakazawa T, Chhajlani V, Petersson M, Karre K, Masucci G, Celis E, Sette A, Southwood S, Appella E, Kiessling R. Synthetic peptides derived from the melanocyte-stimulating hormone receptor MC1R can stimulate HLA-A2-restricted cytotoxic T lymphocytes that recognize naturally processed peptides on human melanoma cells. Cancer Res. 1997 Oct 1;57(19):4348-55.
55. Scardino A, Gross DA, Alves P, Schultze JL, Graff-Dubois S, Faure O, Tourdot S, Chouaib S, Nadler LM, Lemonnier FA, Vonderheide RH, Cardoso AA, Kosmatopoulos K. HER-2/neu and hTERT cryptic epitopes as novel targets for broad spectrum tumor immunotherapy. J Immunol. 2002 Jun 1;168(11):5900-6.
56. Scheibenbogen C, Sun Y, Keilholz U, Song M, Stevanovic S, Asemissen AM, Nagorsen D, Thiel E, Rammensee HG, Schadendorf D. Identification of known and novel immunogenic T-cell epitopes from tumor antigens recognized by peripheral blood T cells from patients responding to IL-2-based treatment. Int J Cancer. 2002 Mar 20;98(3):409-14.
57. Schiavetti F, Thonnard J, Colau D, Boon T, Coulie PG. A human endogenous retroviral sequence encoding an antigen recognized on melanoma by cytolytic T lymphocytes. Cancer Res. 2002 Oct 1;62(19):5510-6.
58. Schmidt SM, Schag K, Muller MR, Weinschenk T, Appel S, Schoor O, Weck MM, Grunebach F, Kanz L, Stevanovic S, Rammensee HG, Brossart P. Induction of adipophilin-specific cytotoxic T lymphocytes using a novel HLA-A2-binding peptide that mediates tumor cell lysis. Cancer Res. 2004 Feb 1;64(3):1164-70.
59. Schmollinger JC, Vonderheide RH, Hoar KM, Maecker B, Schultze JL, Hodi FS, Soiffer RJ, Jung K, Kuroda MJ, Letvin NL, Greenfield EA, Mihm M, Kutok JL, Dranoff G. Melanoma inhibitor of apoptosis protein (ML-IAP) is a target for immune-mediated tumor destruction. Proc Natl Acad Sci U S A. 2003 Mar 18;100(6):3398-403. Epub 2003 Mar 7.
60. Schwitalle Y, Linnebacher M, Ripberger E, Gebert J, von Knebel Doeberitz M. Immunogenic peptides generated by frameshift mutations in DNA mismatch repair-deficient cancer cells. Cancer Immun. 2004 Nov 25;4:14.
61. Slingluff CL Jr, Petroni GR, Yamshchikov GV, Hibbitts S, Grosh WW, Chianese-Bullock KA, Bissonette EA, Barnd DL, Deacon DH, Patterson JW, Parekh J, Neese PY, Woodson EM, Wiernasz CJ, Merrill P. Immunologic and clinical outcomes of vaccination with a multiepitope melanoma peptide vaccine plus low-dose interleukin-2 administered either concurrently or on a delayed schedule. J Clin Oncol. 2004 Nov 15;22(22):4474-85.
62. Spierings E, Brickner AG, Caldwell JA, Zegveld S, Tatsis N, Blokland E, Pool J, Pierce RA, Mollah S, Shabanowitz J, Eisenlohr LC, van Veelen P, Ossendorp F, Hunt DF, Goulmy E, Engelhard VH. The minor histocompatibility antigen HA-3 arises from differential proteasome-mediated cleavage of the lymphoid blast crisis (Lbc) oncoprotein. Blood. 2003 Jul 15;102(2):621-9. Epub 2003 Mar 27.
63. Tourdot S, Scardino A, Saloustrou E, Gross DA, Pascolo S, Cordopatis P, Lemonnier FA, Kosmatopoulos K. A general strategy to enhance immunogenicity of low-affinity HLA-A2. 1-associated peptides: implication in the identification of cryptic tumor epitopes. Eur J Immunol. 2000 Dec;30(12):3411-21.
64. Traversari C, van der Bruggen P, Luescher IF, Lurquin C, Chomez P, Van Pel A, De Plaen E, Amar-Costesec A, Boon T. A nonapeptide encoded by human gene MAGE-1 is recognized on HLA-A1 by cytolytic T lymphocytes directed against tumor antigen MZ2-E. J Exp Med. 1992 Nov 1;176(5):1453-7.
65. Loftus DJ, Castelli C, Clay TM, Squarcina P, Marincola FM, Nishimura MI, Parmiani G, Appella E, Rivoltini L. Identification of epitope mimics recognized by CTL reactive to the melanoma/melanocyte-derived peptide MART-1(27-35). J Exp Med. 1996 Aug 1;184(2):647-57.
66. Steven NM, Annels NE, Kumar A, Leese AM, Kurilla MG, Rickinson AB. Immediate early and early lytic cycle proteins are frequent targets of the Epstein-Barr virus-induced cytotoxic T cell response. J Exp Med. 1997 May 5;185(9):1605-17.

67. Burrows SR, Gardner J, Khanna R, Steward T, Moss DJ, Rodda S, Suhrbier A. Five new cytotoxic T cell epitopes identified within Epstein-Barr virus nuclear antigen 3. J Gen Virol. 1994 Sep;75 (Pt 9):2489-93.
68. Kienzle N, Sculley TB, Poulsen L, Buck M, Cross S, Raab-Traub N, Khanna R. Identification of a cytotoxic T-lymphocyte response to the novel BARF0 protein of Epstein-Barr virus: a critical role for antigen expression. J Virol. 1998 Aug;72(8):6614-20.
69. Khanna R, Sherritt M, Burrows SR. EBV structural antigens, gp350 and gp85, as targets for ex vivo virus-specific CTL during acute infectious mononucleosis: potential use of gp350/gp85 CTL epitopes for vaccine design. J Immunol. 1999 Mar 1;162(5):3063-9.
70. Khanna R, Burrows SR, Nicholls J, Poulsen LM. Identification of cytotoxic T cell epitopes within Epstein-Barr virus (EBV) oncogene latent membrane protein 1 (LMP1): evidence for HLA A2 supertype-restricted immune recognition of EBV-infected cells by LMP1-specific cytotoxic T lymphocytes. Eur J Immunol. 1998 Feb;28(2):451-8.
71. Lee SP, Thomas WA, Murray RJ, Khanim F, Kaur S, Young LS, Rowe M, Kurilla M, Rickinson AB. HLA A2.1-restricted cytotoxic T cells recognizing a range of Epstein-Barr virus isolates through a defined epitope in latent membrane protein LMP2. J Virol. 1993 Dec;67(12):7428-35.
72. Lee SP, Tierney RJ, Thomas WA, Brooks JM, Rickinson AB. Conserved CTL epitopes within EBV latent membrane protein 2: a potential target for CTL-based tumor therapy. J Immunol. 1997 Apr 1;158(7):3325-34.
73. Rehermann B, Ferrari C, Pasquinelli C, Chisari FV. The hepatitis B virus persists for decades after patients' recovery from acute viral hepatitis despite active maintenance of a cytotoxic T-lymphocyte response. Nat Med. 1996 Oct;2(10):1104-8.
74. Nayersina R, Fowler P, Guilhot S, Missale G, Cerny A, Schlicht HJ, Vitiello A, Chesnut R, Person JL, Redeker AG, Chisari FV. HLA A2 restricted cytotoxic T lymphocyte responses to multiple hepatitis B surface antigen epitopes during hepatitis B virus infection. J Immunol. 1993 May 15;150(10):4659-71.
75. Khan N, Cobbold M, Keenan R, Moss PA. Comparative analysis of CD8+ T cell responses against human cytomegalovirus proteins pp65 and immediate early 1 shows similarities in precursor frequency, oligoclonality, and phenotype. J Infect Dis. 2002 Apr 15;185(8):1025-34. Epub 2002 Mar 25.
76. Solache A, Morgan CL, Dodi AI, Morte C, Scott I, Baboonian C, Zal B, Goldman J, Grundy JE, Madrigal JA. Identification of three HLA-A*0201-restricted cytotoxic T cell epitopes in the cytomegalovirus protein pp65 that are conserved between eight strains of the virus. J Immunol. 1999 Nov 15;163(10):5512-8.
77. Nastke MD, Herrgen L, Walter S, Wernet D, Rammensee HG, Stevanovic S. Major contribution of codominant CD8 and CD4 T cell epitopes to the human cytomegalovirus-specific T cell repertoire. Cell Mol Life Sci. 2005 Jan;62(1):77-86.
78. Solache A, Morgan CL, Dodi AI, Morte C, Scott I, Baboonian C, Zal B, Goldman J, Grundy JE, Madrigal JA. Identification of three HLA-A*0201-restricted cytotoxic T cell epitopes in the cytomegalovirus protein pp65 that are conserved between eight strains of the virus. J Immunol. 1999 Nov 15;163(10):5512-8.
79. Shirai M, Arichi T, Nishioka M, Nomura T, Ikeda K, Kawanishi K, Engelhard VH, Feinstone SM, Berzofsky JA. CTL responses of HLA-A2.1-transgenic mice specific for hepatitis C viral peptides predict epitopes for CTL of humans carrying HLA-A2.1. J Immunol. 1995 Mar 15;154(6):2733-42.
80. Shirai M, Okada H, Nishioka M, Akatsuka T, Wychowski C, Houghten R, Pendleton CD, Feinstone SM, Berzofsky JA. An epitope in hepatitis C virus core region recognized by cytotoxic T cells in mice and humans. J Virol. 1994 May;68(5):3334-42.
81. Cerny A, McHutchison JG, Pasquinelli C, Brown ME, Brothers MA, Grabscheid B, Fowler P, Houghton M, Chisari FV. Cytotoxic T lymphocyte response to hepatitis C virus-derived peptides containing the HLA A2.1 binding motif. J Clin Invest. 1995 Feb;95(2):521-30.
82. Scognamiglio P, Accapezzato D, Casciaro MA, Cacciani A, Artini M, Bruno G, Chircu ML, Sidney J, Southwood S, Abrignani S, Sette A, Barnaba V. Presence of effector CD8+ T cells in hepatitis C virus-exposed healthy seronegative donors. J Immunol. 1999 Jun 1;162(11):6681-9.
83. Battegay M, Fikes J, Di Bisceglie AM, Wentworth PA, Sette A, Celis E, Ching WM, Grakoui A, Rice CM, Kurokohchi K, et al. Patients with chronic hepatitis C have circulating cytotoxic T cells which recognize hepatitis C virus-encoded peptides binding to HLA-A2.1 molecules. J Virol. 1995 Apr;69(4):2462-70.

84. Yang OO, Kalams SA, Rosenzweig M, Trocha A, Jones N, Koziel M, Walker BD, Johnson RP. Efficient lysis of human immunodeficiency virus type 1-infected cells by cytotoxic T lymphocytes. J Virol. 1996 Sep;70(9):5799-806.
85. Parker KC, Bednarek MA, Coligan JE. Scheme for ranking potential HLA-A2 binding peptides based on independent binding of individual peptide side-chains. J Immunol. 1994 Jan 1;152(1):163-75.
86. Hadida F, Haas G, Zimmermann N, Hosmalin A, Spohn R, Samri A, Jung G, Debre P, Autran B. CTLs from lymphoid organs recognize an optimal HLA-A2-restricted and HLA-B52-restricted nonapeptide and several epitopes in the C-terminal region of HIV-1 Nef. J Immunol. 1995 Apr 15;154(8):4174-86.
87. Falk K, Rotzschke O, Stevanovic S, Jung G, Rammensee HG. Allele-specific motifs revealed by sequencing of self-peptides eluted from MHC molecules. Nature. 1991 May 23;351(6324):290-6.
88. Harrer T, Harrer E, Kalams SA, Barbosa P, Trocha A, Johnson RP, Elbeik T, Feinberg MB, Buchbinder SP, Walker BD. Cytotoxic T lymphocytes in asymptomatic long-term nonprogressing HIV-1 infection. Breadth and specificity of the response and relation to in vivo viral quasispecies in a person with prolonged infection and low viral load. J Immunol. 1996 Apr 1;156(7):2616-23.
89. Dupuis M, Kundu SK, Merigan TC. Characterization of HLA-A 0201-restricted cytotoxic T cell epitopes in conserved regions of the HIV type 1 gp160 protein. J Immunol. 1995 Aug 15;155(4):2232-9.
90. Tarpey I, Stacey S, Hickling J, Birley HD, Renton A, McIndoe A, Davies DH. Human cytotoxic T lymphocytes stimulated by endogenously processed human papillomavirus type 11 E7 recognize a peptide containing a HLA-A2 (A*0201) motif. Immunology. 1994 Feb;81(2):222-7.
91. Ressing ME, de Jong JH, Brandt RM, Drijfhout JW, Benckhuijsen WE, Schreuder GM, Offringa R, Kast WM, Melief CJ. Differential binding of viral peptides to HLA-A2 alleles. Implications for human papillomavirus type 16 E7 peptide-based vaccination against cervical carcinoma. Eur J Immunol. 1999 Apr;29(4):1292-303.
92. Koelle DM, Chen HB, Gavin MA, Wald A, Kwok WW, Corey L. CD8 CTL from genital herpes simplex lesions: recognition of viral tegument and immediate early proteins and lysis of infected cutaneous cells. J Immunol. 2001 Mar 15;166(6):4049-58.
93. Utz U, Koenig S, Coligan JE, Biddison WE. Presentation of three different viral peptides, HTLV-1 Tax, HCMV gB, and influenza virus M1, is determined by common structural features of the HLA-A2.1 molecule. J Immunol. 1992 Jul 1;149(1):214-21.
94. Falk K, Rotzschke O, Deres K, Metzger J, Jung G, Rammensee HG. Identification of naturally processed viral nonapeptides allows their quantification in infected cells and suggests an allele-specific T cell epitope forecast. J Exp Med. 1991 Aug 1;174(2):425-34.
95. Man S, Newberg MH, Crotzer VL, Luckey CJ, Williams NS, Chen Y, Huczko EL, Ridge JP, Engelhard VH. Definition of a human T cell epitope from influenza A non-structural protein 1 using HLA-A2.1 transgenic mice. Int Immunol. 1995 Apr;7(4):597-605.
96. Kennedy R, Poland GA. T-Cell epitope discovery for variola and vaccinia viruses. Rev Med Virol. 2006;17(2): 93-113.
96 Kennedy R, Poland GA. T-Cell epitope discovery for variola and vaccinia viruses. Rev Med Virol. 2006;17(2): 93-113.
97. Ingo Drexler I, Caroline Staib C, Wolfgang Kastenmuller W, Stefan Stevanovic S, Burkhard Schmidt B, Francois A. Lemonnier FA, Hans-Georg Rammensee HG, Dirk H. Busch DH, Helga Bernhard H, Volker Erfle V, Gerd Sutter G. Identification of vaccinia virus epitope-specific HLA-A*0201-restricted T cells and comparative analysis of smallpox vaccines. Proc Natl Acad Sci U S A. 2003; 100(1):217-222.
98. Du Pasquier RA, Kuroda MJ, Schmitz JE, Zheng Y, Martin K, Peyerl FW, Lifton M, Gorgone D, Autissier P, Letvin NL, Koralnik IJ. Low frequency of cytotoxic T lymphocytes against the novel HLA-A*0201-restricted JC virus epitope VP1(p36) in patients with proven or possible progressive multifocal leukoencephalopathy. J Virol. 2003 Nov;77(22):11918-26.
